# Supplementary material for: SARS-CoV-2 Genetic Variants Identified in Selected Regions of Ethiopia Through Whole Genome Sequencing: Insights from the Fifth Wave of COVID-19
Source: Genes (Basel). 2025 Mar 18;16(3):351. doi: 10.3390/genes16030351 (PMC11942139; doi:10.3390/genes16030351)
Supplement: Supplementary file 1 [file genes-16-00351-s001.zip › genes-3501442-supplementary.pdf]

Table S1. SARS-CoV-2 nucleotide mutations in different genes position during the fifth wave the pandemic in Ethiopia.

Gene	Position	Mutation
S	97, 421-426, 445, 455, 457, 462, 465, 486, 500, 658, 720, 737-742, 744-746, 763-765	S:K97R, S:K97R, S:Y423L, S:K424stop, S:L425I, S:P426T, S:Y421S, S:V445A, S:L455S, S:L455S, S:R457G, S:K462Q, S:E465X, S:F486V, S:F486V. S:F486V, S:T500S, S:N658S, S:N658S, S:I720X, S:D737V, S:C738V, S:T739Q, S:M740C, S:Y741T, S:I742F, S:G744W, S:D745stop, S:S746F, S:N764E, S:L763I, S:R765S, S:N764X, S:R765X.
M	3, 83, 151, 154, 164, 167-169, 198-199, 203-206, 212-213.	M:D3N, M:D3N, M:D3N, M:D3N, M:D3N, M:D3N, M:D3N, M:D3N, M:D3N, M:D3N, M:D3N, M:A83T, M:I151M, M:I151M, M:I151M, M:H154D, M:L164M, M:Y178X, M:E167A, M:E167K, M:Y179stop, M:R198G, M:Y199C, M:L206stop, M:N203X, M:Y204F, M:K205A, M:N203T, M:Y204N, M:K205A, M:L206stop, M:S212X, M:S212X, M:S213X, M:S213X, M:S212X, M:S212X, M:S212R, M:S213X, M:S213X, M:S212X.
N	379	N:T379N
Nsp1	7	nsp1:G7X
Nsp8	50, 74, 167 and 177	nsp8:D50N, nsp8:A74S, nsp8:A74S, nsp8:A74S, nsp8:A74S, nsp8:A74S, nsp8:A74S, nsp8:V167F, nsp8:S177L
Nsp9	91	nsp9:I91V
Nsp10	75	nsp10:L75M
Nsp13	512	nsp13:V521I
ORF3a	92, 213, 275	ORF3a:S92P, ORF3a:Q213R, ORF3a:L275F
ORF7a	48	ORF7a:P48A
Plpro	684, 906, 932, 1234	PLpro:I684V, PLpro:E906A, PLpro:V932L, PLpro:V1234E
RdRp	930	RdRP:V930I
